# The effects of body direction and posture on taking the perspective of a humanoid avatar in a virtual environment

**DOI:** 10.1371/journal.pone.0261063

**Published:** 2021-12-21

**Authors:** Sachiyo Ueda, Kazuya Nagamachi, Junya Nakamura, Maki Sugimoto, Masahiko Inami, Michiteru Kitazaki

**Affiliations:** 1 Department of Computer Science and Engineering, Toyohashi University of Technology, Toyohashi, Aichi, Japan; 2 Department of Information and Computer Science, Keio University, Yokohama, Kanagawa, Japan; 3 Research Center for Advanced Science and Technology, The University of Tokyo, Meguro-ku, Tokyo, Japan; University College Dublin, IRELAND

## Abstract

Visual perspective taking is inferring how the world looks to another person. To clarify this process, we investigated whether employing a humanoid avatar as the viewpoint would facilitate an imagined perspective shift in a virtual environment, and which factor of the avatar is effective for the facilitation effect. We used a task that involved reporting how an object looks by a simple direction judgment, either from the avatar’s position or from the position of an empty chair. We found that the humanoid avatar’s presence improved task performance. Furthermore, the avatar’s facilitation effect was observed only when the avatar was facing the visual stimulus to be judged; performance was worse when it faced backwards than when there was only an empty chair facing forwards. This suggests that the avatar does not simply attract spatial attention, but the posture of the avatar is crucial for the facilitation effect. In addition, when the directions of the head and the torso were opposite (i.e., an impossible posture), the avatar’s facilitation effect disappeared. Thus, visual perspective taking might not be facilitated by the avatar when its posture is biomechanically impossible because we cannot embody it. Finally, even when the avatar’s head of the possible posture was covered with a bucket, the facilitation effect was found with the forward-facing avatar rather than the backward-facing avatar. That is, the head/gaze direction cue, or presumably the belief that the visual stimulus to be judged can be seen by the avatar, was not required. These results suggest that explicit perspective taking is facilitated by embodiment towards humanoid avatars.

## Introduction

Visual perspective taking, or when humans move their virtual viewpoint to another person’s perspective [1], may be a basis for more sophisticated social abilities such as empathy [2–4], shared intentionality [5], and theory of mind [6]. Visual perspective taking is not a unitary ability but can be divided into different levels [1, 7, 8]. Level 1 is the ability to understand whether an object is visible to others, and Level 2 is the ability to understand others’ perspectives of the object. Level 2 perspective taking develops later [1, 9, 10], which suggests that it involves cognitively demanding computational processing. Moreover, visual perspective taking can be divided into two different systems: implicit perspective taking and explicit perspective taking [8, 11]. Implicit perspective taking occurs when participants are asked to answer only from the egocentric perspective or the first-person perspective; their responses are affected by the presence of another person in the scene. Explicit perspective taking requires participants to take another’s perspective, and respond from their viewpoint.

Currently, it is not very clear how visual perspective taking works. There is debate over whether implicit visual perspective taking is automatic and spontaneous [12–16], or if it includes a conscious process [17–20]. Samson et al. [12] showed that incongruent information from another person’s perspective interferes with performance in the Level 1 visual perspective taking task, even when the participant is ignoring that other person (altercentric intrusions). Tversky and Hard [21] showed that, in the task of describing the spatial relationship between two objects (Level 2 perspective taking), the presence of another person increased the spontaneous adoption of that person’s point of view. They also found that when asking a question drawing attention to action, more participants adopted the other person’s perspective than their own. Surtees et al. [14] showed that performance in the Level 2 visual perspective taking task is spontaneously affected by another person in an interactive task. Ward et al. [15] used a task involving the classic mental rotation task, and showed that Level 2 perspective taking occurred spontaneously through the presence of agents even though the agents were irrelevant to the task. They demonstrated that the reaction time (RT) to judge letters presented on a table at various angles was shorter, not only when the rotation angle as viewed by the observer was small, but also when the rotation angle as viewed by another person (humanoid avatar) was small. The facilitation effect was enhanced when participants were asked to explicitly take the perspective of the avatar. This facilitation effect disappeared when a lamp (inanimate object) was presented instead of the humanoid avatar. They called it “perceptual simulation” of others’ viewing perspective [15]. In a following study, Ward et al. [16] reported that the facilitation effect of another person does not require that person to be looking at an object. They concluded that visual perspective taking is a general spatial-navigational ability that is influenced by the spatial location of another person, but not their gaze. However, Cole et al. [17] demonstrated a perspective taking effect even when the agent could not see the same stimuli as the participant due to a barrier, and concluded that humans do not spontaneously take the perspective of others. Kuhn et al. [18] showed that when participants are given ample time to explore the visual environment, gaze following is modulated by another person’s visual perspective; participants fixate on a target object faster when the agent can see the object than when it is occluded from the agent’s sight. On the other hand, when participants are asked to rapidly discriminate a target, their performance is not modulated by another person’s visibility. Thus, they concluded that visual perspective taking is not automatic [18]. Samuel et al. [20] showed that visual perspective taking is not perceptual simulation, but a conscious process to solve a problem by using naïve ideas about how vision works.

In explicit perspective taking, participants mentally rotate or transform their own perspective to another person’s position in the Level 2 perspective taking task, but not in the Level 1 perspective taking task [7, 22]. An imagined shift of perspective to an arbitrary position is possible even when the physical body of another person is not present [23–27]. However, the performance of the Level 2 perspective taking task was better when a doll or an avatar, was presented compared to a non-human object [7, 22]. The embodiment of the agent can be a facilitation factor in explicit Level 2 perspective taking [22, 28]. Kessler and Thomson [22] showed that congruence between participants’ body posture and direction of mental self-rotation improves visual perspective taking. Since this effect did not disappear, even in the absence of an avatar (empty chair), they argued that the embodiment effect in perspective taking mainly depends on a self-initiated emulation of a body rotation. However, at the same time, they showed that the posture of the avatar could not be fully ignored, although it was irrelevant to the task. Vestibular stimulation of participants impairs performance of visual perspective taking [29]. Thus, there may be two critical factors for explicit visual perspective taking: the endogenous (i.e., the participant’s) motoric or vestibular embodiment and the exogenous (i.e., the avatar’s) embodiment [22, 29].

In the current study, we aimed to investigate which features of a humanoid avatar facilitate explicit Level 2 perspective taking, to understand the effect of exogenous embodiment. We developed a virtual reality task involving a simple direction judgment to examine how a humanoid avatar facilitates visual perspective taking in a virtual environment. In Experiment 1, two conditions were created: one with an avatar sitting in a chair and looking at a visual stimulus and another with only the empty chair. Participants were asked to judge the direction of the gap in a circle (i.e., a Landolt ring) from the avatar’s perspective or the empty chair’s position. Thus, participants were explicitly asked to take the visual perspective of the location of the avatar or chair. We then examined whether the presentation of the avatar facilitated the perspective transformation. We also manipulated the interval between the presentation of the avatar or the chair and the visual stimulus to examine the time scale of perspective taking.

In Experiment 2, we manipulated the orientation of the avatar with respect to the target stimulus and examined whether the avatar’s gaze on the target was necessary for Level 2 perspective taking. If the RT was shortened only when the avatar’s body was directed at the target (i.e., in the forward-facing condition), it would indicate that the humanoid avatar is not simply facilitating the perspective judgment from an arbitrary position by strongly attracting attention, but rather that the avatar’s gaze and/or body posture must necessarily match the orientation in which the target can be seen by it.

In Experiment 3, we introduced an impossible-posture avatar whose head was oriented in the opposite direction to the torso to test whether the combination of the head and the torso directions could affect the facilitation effect on visual perspective taking.

In Experiment 4, we investigated whether the head or the torso was crucial for the facilitation effect by covering the avatar’s head with a bucket. If the head direction is critical, the RT would not be shortened when the avatar, with a bucket covering its head, is facing the target. Instead, if the direction of the torso is crucial, the RT will be shorter when the avatar is facing the target, irrespective of whether a bucket is covering the avatar’s head or not.

## Experiment 1

### Methods

#### Participants

Twenty paid volunteers participated in the experiment (17 men, 3 women, all aged 19–24 years). The sample size was determined by our previous experiences before conducting the experiments. It corresponded to an effect size f of 0.195, alpha = 0.05, power = 0.8 using the G*Power 3.1 [30, 31]. Participants were undergraduate and graduate students of Toyohashi University of Technology. All had normal or corrected-to-normal vision and were naïve to the purpose of the study.

In all the studies, participants provided written informed consent before the experiment. All the experiments were approved by the Ethical Committee for Human-Subject Research of Toyohashi University of Technology, and were performed in accordance with the committee’s guidelines and regulations.

#### Apparatus

The visual stimuli were generated and controlled by a computer using Unity 2018 .1.6f1 and presented on a head-mounted display (HTC Vive Pro: 1,440 × 1,600 pixels, 90 Hz refresh). The participants responded in the task by moving a joystick. We used Unity 2018 and its C# scripts to generate stimuli and control the experiments. The refresh rate and the sampling rate of responses were 90Hz synchronously. The system delay was less than 11 ms.

#### Stimuli and conditions

**Fig 1:**
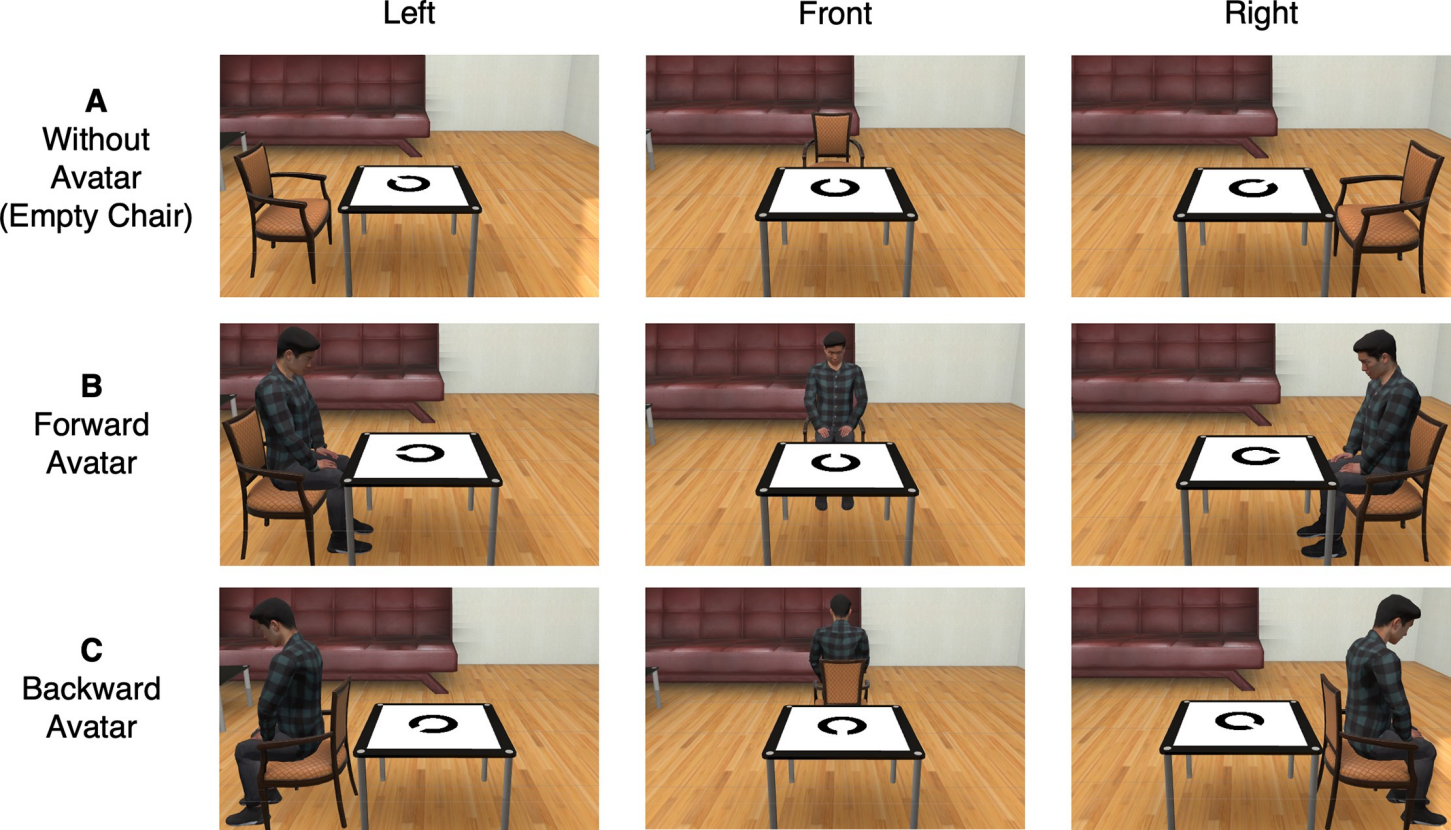
A subset of the stimuli used in the experiments. An empty chair (A) or a forward avatar (B) was presented in either the left, front, or right position from the participant’s point of view in Experiment 1. In addition to the avatar presentation conditions in Experiment 1, there was a backward avatar condition in which the avatar was sitting backwards against a desk (C) in Experiment 2. Participants were asked to judge the direction of the gap in the circle from the avatar’s perspective or the empty chair’s position and to respond with the joystick as accurately and quickly as possible.

In the virtual space, a table was in the center of a room, and either an empty chair or an avatar sitting in a chair was presented in one of three positions, that is, on the left, right, or other side of the table, based on the participant’s perspective (Fig 1A and 1B). Then, a broken circle was presented on the table. The gap in the broken circle was angled in one of eight directions (0°, 45°, 90°, 135°, 180°, 225°, 270°, and 315°), like a Landolt ring. We created two conditions for the interval between the presentation of the chair or avatar and the presentation of the circle (short: 200 ms; long: 1,000 ms). There were 96 combinations of trials (2 with/without the avatar, 2 short/long intervals, 3 positions of the avatar and chair, and 8 directions of the gap in the circle). The directions of the gap in the circle were merged in the analysis. During all experiments, the participant’s own body was not presented in the virtual space (i.e., even if the participants looked down, they could not see their virtual self-bodies).

#### Procedure

**Fig 2:**
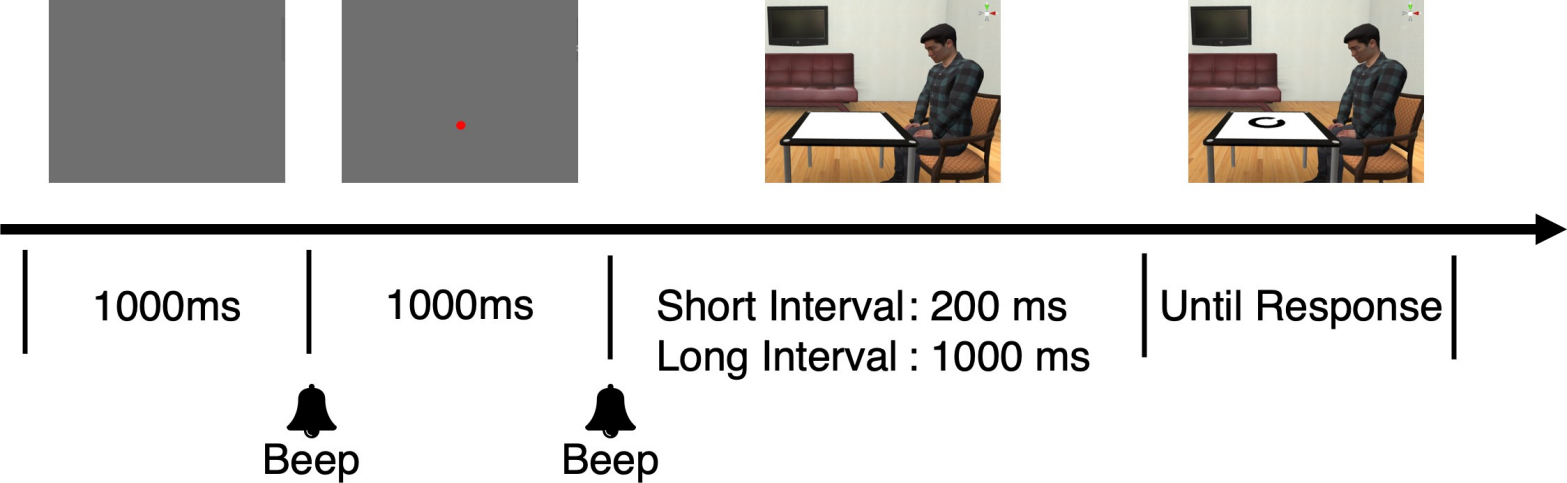
Procedure of Experiment 1.

Each trial began with a blank black screen for 1,000 ms, which was followed by a red fixation dot. Then, 1,000 ms later, the fixation dot disappeared and the room with the table, chair, and/or avatar appeared. After 200 ms (short interval) or 1,000 ms (long interval), the broken circle was presented on the table. The participants were asked to judge the direction of the gap in the circle from the avatar’s perspective or the empty chair’s position and to respond with the joystick as accurately and quickly as possible (Fig 2). If the gap direction was 135° counterclockwise from the participants’ point of view (when the 6 o’clock position is defined as 0°), as in Fig 2, the participant’s task was to adjust the direction from the perspective of the avatar, that is, the joystick should have been moved to the 45° counterclockwise position. Participants received no feedback. The next trial began immediately after the joystick response.

Before the practice trials, a direction judgment task from the participant’s perspective was conducted. Then, 12 practice trials (2 with/without the avatar, 2 short/long intervals, and 3 positions of the avatar and chair) were presented, and the participants judged the direction of the gap in the circle from the avatar’s perspective or the empty chair’s position.

In each test session, all 96 combinations of the conditions were repeated twice in a random order, for a total of 192 trials. One session lasted about 13 minutes. Each participant completed four test sessions, for a total of 768 test trials. It took approximately 90 minutes for each participant to finish this experiment, including the time required to provide the experimental instructions, to conduct the practice trials and the test sessions, and to take breaks between sessions to avoid fatigue.

#### Data analysis

Individual mean RTs and error rates were calculated for each of the twelve conditions (i.e., with/without the avatar, short/long, and left/front/right position of the avatar/chair). For the analysis, we treated the trials in which the participant moved the joystick within the range of ± 22.5° from the correct angle, as the correct response. RTs were determined as the time from the onset of the broken circle to the time when the joystick reached the end position (i.e., 2.5 cm from the center of the joy stick). Trials for which the RT was shorter than 150 ms (0%) and trials for which the RT was longer than three standard deviations from the mean RT of each condition for each participant (1.5%) were excluded as outliers from the analysis. Trials in which the participants made an error were also excluded from the RT analysis (approximately 6.8% of the trials). The RTs and error rates were submitted to a 2 × 2 × 3 repeated-measures analysis of variance (ANOVA) with the avatar presence, interval, and position of the avatar and chair, as the within-subject factors. If there was a lack of sphericity, the reported values were adjusted using the Greenhouse-Geisser correction [32]. When performing the multiple comparisons after the ANOVAs, we reported the *p*-values that were corrected using Shaffer’s modified sequentially rejective Bonferroni procedure [33].

### Results

The ANOVA of RTs showed significant main effects of the presence of the avatar, *F*(1, 19) = 19.570, *p* < 0.001, η_p_^2^ = 0.507, the length of the interval, *F*(1, 19) = 73.376, *p* < 0.001, η_p_^2^ = 0.794, and the position of the avatar and chair, *F*(1.170, 22.222) = 7.864, *p* = 0.001, η_p_^2^ = 0.293. There was also a significant interaction between the avatar presence and interval, *F*(2, 38) = 7.355, *p* = 0.014, η_p_^2^ = 0.279, and between position and interval, *F*(2, 38) = 12.068, *p* < 0.001, η_p_^2^ = 0.388. The avatar presence × position interaction, *F*(2, 38) = 3.138, *p* = 0.055, η_p_^2^ = 0.142, and avatar presence × interval × position interaction, *F*(1.351, 25.670) = 0.383, *p* = 0.604, η_p_^2^ = 0.020, were not significant.

**Fig 3:**
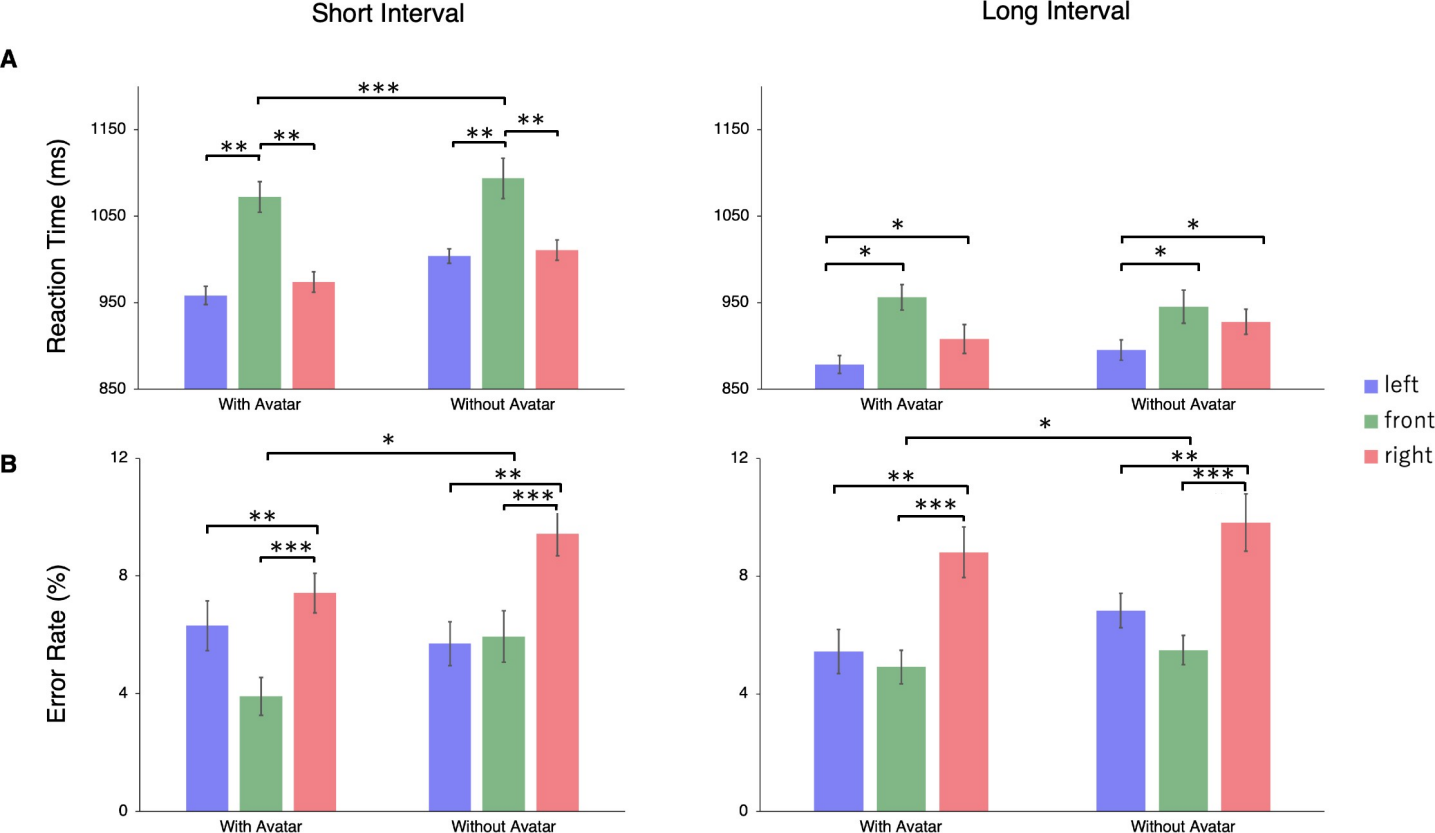
Results of Experiment 1. (A) Mean reaction times (RTs). Three-way repeated measures ANOVA and Shaffer’s post hoc tests were conducted (*p < .05, **p < .01, ***p < .001). The RTs were significantly faster for the “with avatar” condition than the “without avatar” condition, but only in the short interval condition. In the short interval condition, the RTs were slower in the front position than in the other two positions. In the long interval condition, the RTs were faster in the left position than in the other two positions. (B) Error rates. The participants were more accurate when the avatar was present than when it was not. The responses were more accurate when the avatar was in the front and left positions than in the right position. Error bars represent 95% within-subject confidence intervals [34].

Participants’ RTs were significantly faster for the “with avatar” condition than the “without avatar” condition, only in the short interval condition (*p* < 0.001) (Fig 3A). In the short interval condition, the RTs were slower in the front position than in the other two positions (*ps* < 0.01). In the long interval condition, the RTs were faster in the left position than in the other two positions (*ps* < 0.05). In all conditions, the long interval condition had faster RTs than the short interval condition.

The ANOVA of error rates revealed a significant main effect of the presence of the avatar, *F*(1, 19) = 7.335, *p* = 0.014, η_p_^2^ = 0.279 (Fig 3B). The participants were more accurate when the avatar was present than when it was not. The main effect of position was also significant, *F*(2, 38) = 11.696, *p* < 0.001, η_p_^2^ = 0.381. Participants responded more accurately when the avatar was in the front and left positions than in the right position. No other main effect or interactions were found to be significant.

### Discussion

The humanoid avatar was associated with improved performance of identifying the orientation of a visual stimulus from an imagined position only in the short interval condition (200 ms). When the avatar was present, the participant’s viewpoint moved quickly to the position of the avatar, but this process either did not occur or took a long time in the chair condition. In the long interval condition, participants may have had enough time to transform the viewpoint to an arbitrary position regardless of the presence of the avatar.

This facilitation effect of the humanoid avatar was basically consistent with previous findings [7, 15, 22]. These studies also showed that the presence of a humanoid avatar makes it faster to determine how the visual stimulus looks from that position than it does with an inanimate object [7, 15, 22]. However, one may argue that the results were obtained because humanoid avatars capture attention more easily than an empty chair. To examine this, in Experiment 2, we added a condition in which the humanoid avatar was sitting backwards. We hypothesized that if there is no facilitation effect on the RT in the backward avatar condition, then the embodiment of the avatar is important for efficient perspective taking.

We found that there was also a significant difference in RT depending on the position of the avatar. In the short interval condition, the RTs were longer in the front position than in the right and left positions, and in the long interval condition, the RTs were longer in the front and right positions than in the left position. The tendency that a large angular disparity between avatar position and observer results in a long reaction time was the same with or without an avatar, and is consistent with the previous findings that perspective-taking requires mental self-rotation (e.g., [7, 22]). However, it should be noted that, for the error rate, the performance was most accurate in the front positions. This indicates that the long RT in the front position may involve a trade-off with accuracy. This possibility will be discussed again in the general discussion section.

## Experiment 2

### Methods

#### Participants

Twenty paid volunteers participated in the experiment (15 men, 5 women, all aged 20–24 years). Eight of them had participated in Experiment 1. The sample size was determined by our previous experiences before conducting the experiments. It corresponded to an effect size f of 0.21, alpha = 0.05, power = 0.8 using the G*Power 3.1 [30, 31]. The participants all had normal or corrected-to-normal vision and were naïve to the purpose of the study.

#### Apparatus

The same apparatus used in Experiment 1 was used in Experiment 2.

#### Stimuli and conditions

This experiment differed from Experiment 1 in that we added the backward avatar condition (Fig 1C). Since the avatar’s facilitation effect was obtained only in the short interval condition in Experiment 1, only the short interval was employed in Experiment 2. There were 72 combinations of trials (3 types of avatar: no avatar, forward avatar, and backward avatar; 3 positions of the avatar and chair; and 8 directions of the gap in the circle). The directions of the gap in the circle were combined in the analysis.

#### Procedure

The procedure was the same as in Experiment 1, but the experimental conditions were changed. In the backward avatar condition, participants were instructed to imagine the perspective when looking from the avatar’s position toward the center of the table. Prior to the practice trials, the direction judgment task from the participant’s perspective was conducted. Then, a block of nine practice trials (3 types of avatar and 3 positions of the avatar and chair) was presented.

In each test session, all 72 combinations of the conditions (3 types of avatar, 3 positions of the avatar and chair, and 8 directions of the gap in the circle) were repeated twice in a random order, for a total of 144 trials. Each participant completed four test sessions, for a total of 576 test trials. It took approximately 60 minutes for each participant to finish this experiment.

#### Data analysis

Individual mean RTs and error rates were calculated for each of the nine conditions (i.e., without/forward/backward avatar and left/front/right position) as in Experiment 1. Trials for which RT was shorter than 150 ms (0%), trials for which RT was longer than three standard deviations from the mean RT (1.6%), and error trials (6.2%) were excluded as outliers from the RT analysis. The RTs and error rates were submitted to a 3 × 3 repeated-measures ANOVA with the avatar type and position of the avatar and chair as the within-subject factors. When conducting the multiple comparisons after the ANOVAs, we reported the *p*-values that were corrected using Shaffer’s modified sequentially rejective Bonferroni procedure [33].

### Results

**Fig 4:**
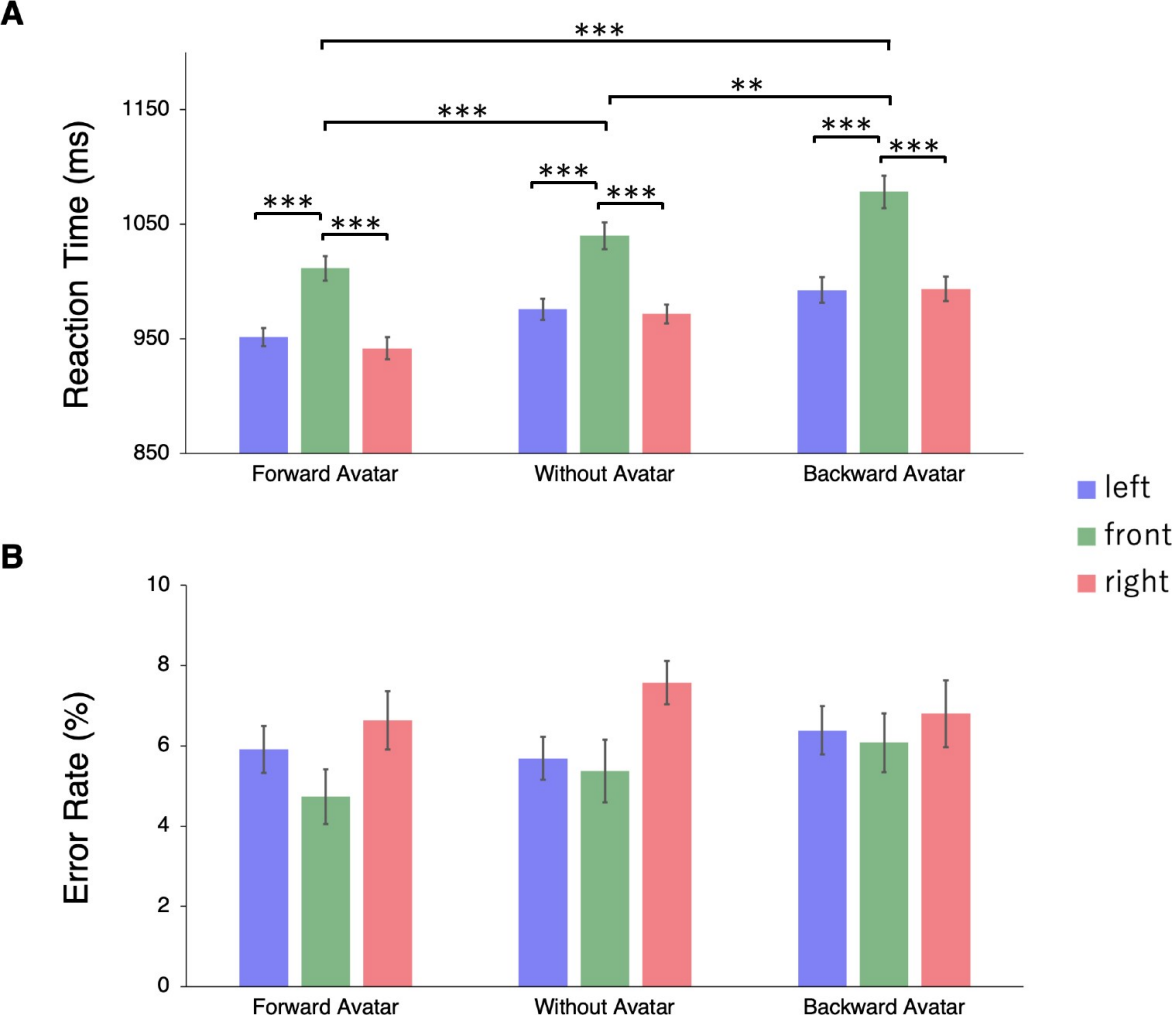
Results of Experiment 2. (A) Mean reaction times (RTs). Two-way repeated measures ANOVA and Shaffer’s post hoc tests were conducted (*p < .05, **p < .01, ***p < .001). The RTs were significantly faster in the order of the forward avatar condition, without avatar condition, and backward avatar condition. The RTs were significantly slower in the front position condition than in the right position and left position conditions. (B) Error rates. There were no significant main effects or interactions for the accuracy. Error bars represent 95% within-subject confidence intervals.

The ANOVA of RTs showed significant main effects of the type of avatar, *F*(2, 38) = 25.459, *p* < 0.001, η_p_^2^ = 0.573, and position of the avatar and chair, *F*(2, 38) = 14.952, *p* < 0.001, η_p_^2^ = 0.440. The avatar presence × position interaction was not significant, *F*(4, 76) = 1.113, *p* = 0.357, η_p_^2^ = 0.055. The RTs were significantly faster in the order of the forward avatar condition, without avatar condition, and backward avatar condition (*ps* < 0.01). The RTs were significantly slower in the front position condition than in the right position and left position conditions (*ps* < 0.001) (Fig 4A). The ANOVA of error rates revealed that there were no significant main effects or interactions for the accuracy (Fig 4B).

### Discussion

We found that the facilitation of the perspective transformation occurred only when the avatar’s body and head were facing the visual stimulus. Thus, the direction of a humanoid avatar’s head and body is an effective cue for the facilitation effect of spatial cognition in perspective taking. This suggests that the facilitation effect of the presence of humanoid avatars on Level 2 perspective taking is not based on attention capture due to the saliency of the humanoid avatars, but on the efficiency of the cognitive process of perspective taking.

In contrast, Ward et al. [16] showed that the facilitation effect of perspective taking exists even when the avatar does not look at an object. This difference in the findings may be due to the fact that our avatar was placed facing away from the object with a full body including the head, but the avatar in Ward et al.’s [16] study averted its gaze while its torso faced the object. Thus, the direction of the torso may be more important than the gaze direction. To investigate this, in Experiment 3, we tested whether the combinations of the head and the torso directions could affect the facilitation effect on visual perspective taking by employing an avatar in an impossible posture.

## Experiment 3

### Methods

#### Participants

Twenty paid volunteers participated in the experiment (17 men, 3 women, all aged 20–23 years). None had participated in either Experiment 1 or 2. The sample size was determined by our previous experiences before conducting the experiments. It corresponded to an effect size f of 0.22, alpha = 0.05, power = 0.8 using the G*Power 3.1 [30, 31]. The participants all had normal or corrected-to-normal vision and were naïve to the purpose of the study.

#### Apparatus

The same apparatus used in Experiments 1 and 2 were used in Experiment 3.

#### Stimuli and conditions

**Fig 5:**
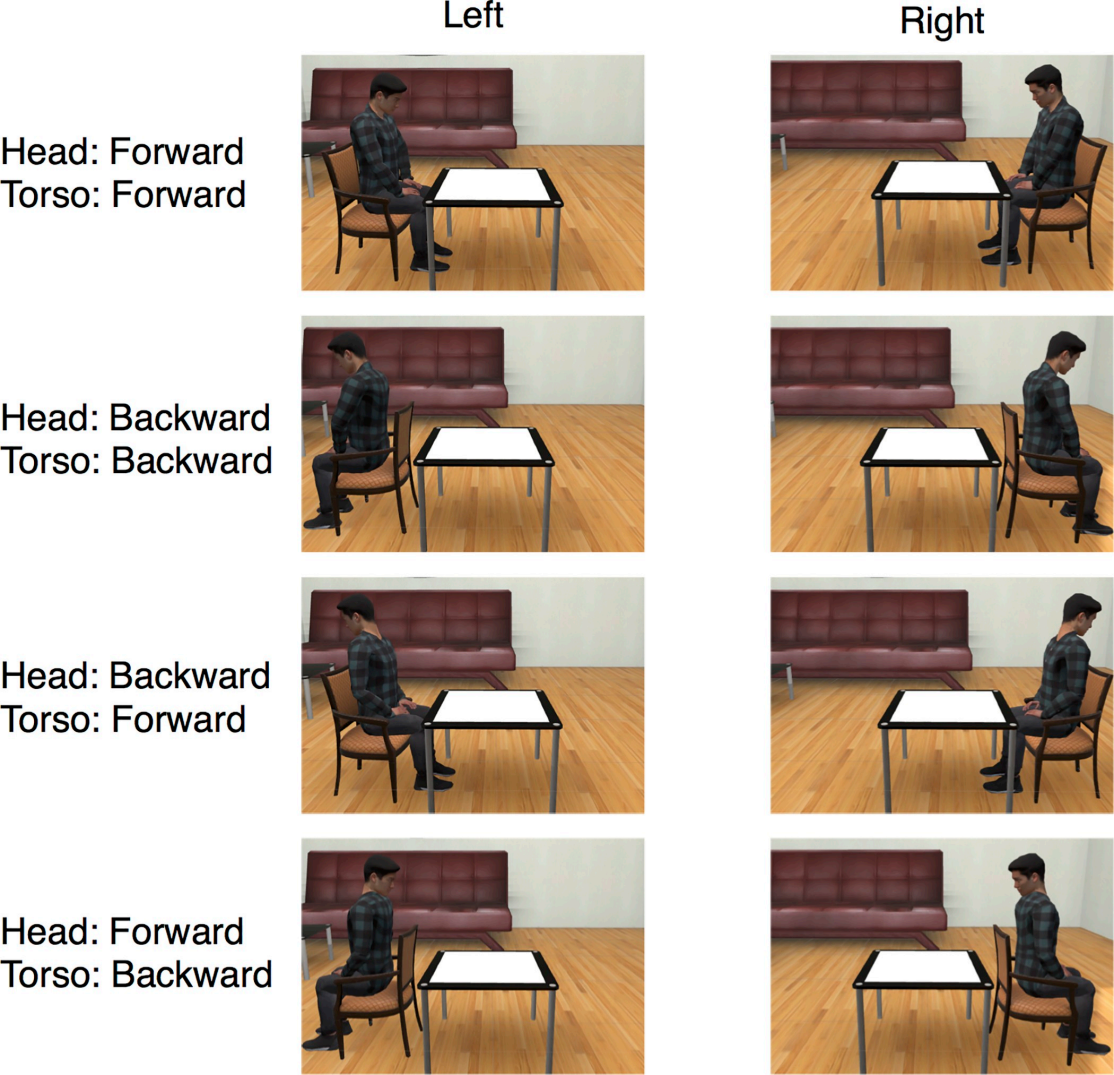
A subset of the stimuli used in Experiment 3. An avatar sitting in a chair was presented in either the left or right position from the participant’s point of view. In addition to the forward avatar and the backward avatar, a torso-only forward avatar and a head-only forward avatar were employed.

In this experiment, we added the avatar whose head was oriented in the opposite direction to the torso (Fig 5). There were 4 conditions: (1) both the head and the torso were facing toward the stimulus, (2) both the head and the torso were facing away from the stimulus, (3) the torso was facing toward the stimulus while the head was facing away, (4) the head was facing toward the stimulus while the torso was facing away. The direction of the chair was always the same as that of the torso. The short interval (200 ms) was employed. The gap in the broken circle was angled in one of four directions (45°, 135°, 225°, and 315°). The avatar sitting in the chair was presented either on the left or right side of the table. There were 32 combinations of trials (4 types of avatar; 2 positions of the avatar and chair; and 4 directions of the gap in the circle). The directions of the gap in the circle were combined in the analysis.

#### Procedure

The procedure was the same as in Experiments 1 and 2, but the experimental conditions were changed. Before the practice trials, the direction judgment task from the participant’s perspective was conducted. Then, a block of eight practice trials (4 types of avatar and 2 positions of the avatar and chair) was presented.

In each test session, all 32 combinations of the conditions (4 types of avatar, 2 positions of the avatar and chair, and 4 directions of the gap in the circle) were repeated twice in a random order, for a total of 64 trials. Each participant completed four test sessions, for a total of 256 test trials. It took approximately 40 minutes for each participant to finish this experiment, including the time required to provide the experimental instructions, take a break, and conduct the practice trials.

#### Data analysis

Individual mean RTs and error rates were calculated for each of the eight conditions using the same procedure as in Experiment 1 and 2. Trials for which the RT was shorter than 150 ms (0%), trials for which the RT was longer than three standard deviations from the mean RT (1.5%), and error trials (13.6%) were excluded as outliers from the RT analysis. The RTs and error rates were submitted to a 4 × 2 repeated-measures ANOVA with the avatar type and position of the avatar and chair as the within-subject factors. If there was a lack of sphericity, the reported values were adjusted using the Greenhouse-Geisser correction [32]. When conducting the multiple comparisons after the ANOVAs, we reported the *p*-values that were corrected using Shaffer’s modified sequentially rejective Bonferroni procedure [33].

### Results

**Fig 6:**
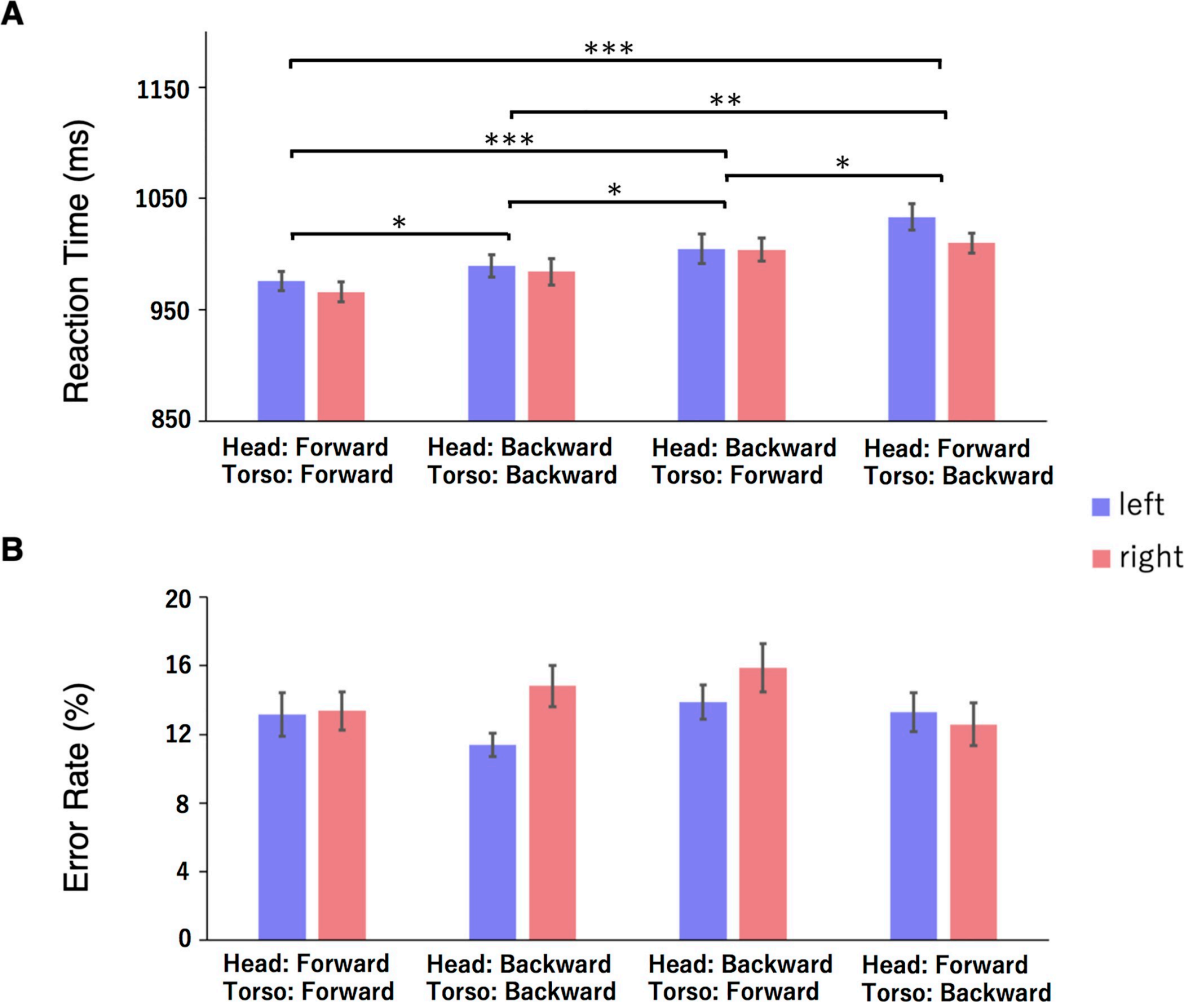
Results of Experiment 3. (A) Mean reaction times (RTs). Two-way repeated measures ANOVA and Shaffer’s post hoc tests were conducted (*p < .05, **p < .01, ***p < .001). The RTs were significantly faster in the order of the condition (1) both the head and the torso were facing forward, (2) both the head and the torso were facing backward, (3) the torso was facing toward the stimulus while the head was facing away, and (4) the head was facing toward the stimulus while the torso was facing away. (B) Error rates. There were no significant main effects or interactions for the accuracy. Error bars represent 95% within-subject confidence intervals [34].

The ANOVA of RTs showed significant main effects regarding the type of avatar, *F*(1.820, 34.574) = 15.450, *p* < 0.001, η_p_^2^ = 0.448. The main effects of the position of the avatar and the interaction were not significant. The RTs were fastest in condition 1, followed by conditions 2, 3, and 4 (*ps* < 0.05, Fig 6A). The ANOVA of error rates revealed that there were no significant main effects or interactions for the accuracy (Fig 6B).

### Discussion

We found that the facilitation of the perspective transformation disappeared when the avatar’s head was oriented in the opposite direction to the torso. The RTs for the impossible-posture avatar conditions were slower than the full-body backward avatar. This suggests that visual perspective taking might not be facilitated by the avatar when its posture is biomechanically impossible. As for the impossible-posture avatars, the torso-only forward condition was responded to faster than the head-only forward condition. Thus, the direction of the torso may be more effective for visual perspective taking than the direction of the head or gaze.

Amorim, Isableu, & Jarraya [35] showed that the facilitation effect of the mental rotation of objects by body analogy is disrupted when the posture cannot be emulated by the sensorimotor system, such as with the impossible postures. Our results are consistent with their findings. Thus, the impossible posture prevents motoric embodiment. Because of this, it was difficult to interpret the pure effect of the direction of the head and the torso from the results of Experiment 3. Thus, we manipulated the direction of the possible-posture avatar with respect to the target, and compared the case where the head was covered with a bucket and the case where it was not, in Experiment 4.

## Experiment 4

### Methods

#### Participants

Twenty paid volunteers participated in the experiment (19 men, 1 woman, all aged 18–25 years). None had participated in either Experiment 1, 2, or 3. The sample size was determined by our previous experiences prior to conducting the experiments. It corresponded to an effect size f of 0.22, alpha = 0.05, power = 0.8 using the G*Power 3.1 [30, 31]. The participants all had normal or corrected-to-normal vision and were naïve to the purpose of the study.

#### Apparatus

The same apparatus used in Experiments 1, 2, and 3 were used in Experiment 4.

#### Stimuli and conditions

**Fig 7:**
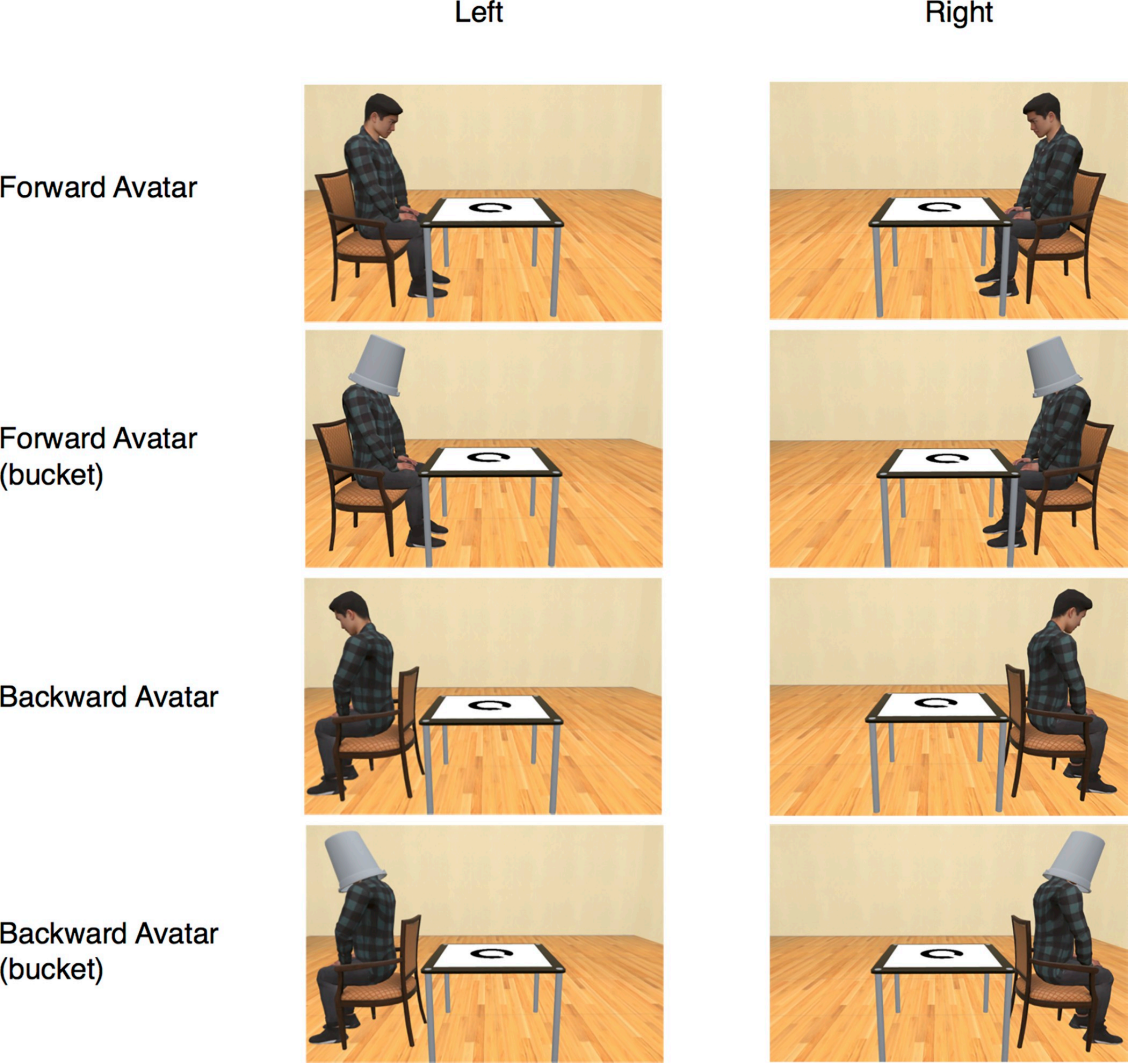
A subset of the stimuli used in Experiment 4. An avatar sitting in a chair was presented in either the left or right position from the participant’s point of view. In addition to the forward avatar and the backward avatar, the forward avatar and the backward avatar with head covered by a bucket were employed.

These were the same as in Experiment 3, except for the avatar conditions. The sofa was removed from the scene. There were four avatar conditions: (1) forward avatar, (2) forward avatar with bucket over its head, (3) backward avatar, and (4) backward avatar with bucket over its head (Fig 7). There were 32 combinations of trials (4 types of avatar; 2 positions of the avatar and chair; and 4 directions of the gap in the circle). The directions of the gap in the circle were combined in the analysis.

#### Procedure

The procedure was the same as in Experiment 3, but the experimental conditions were changed. Before the practice trials, the direction judgment task from the participant’s perspective was conducted. Then, a block of eight practice trials (4 types of avatar and 2 positions of the avatar and chair) was presented twice.

In each test session, all 32 combinations of the conditions (4 types of avatar, 2 positions of the avatar and chair, and 4 directions of the gap in the circle) were repeated twice in random order, for a total of 64 trials. Each participant completed four test sessions, for a total of 256 test trials.

#### Data analysis

Individual mean RTs and error rates were calculated for each of the eight conditions using the same procedure as in Experiments 1–3. Trials for which RT was shorter than 150 ms (0%), trials for which RT was longer than three standard deviations from the mean RT (1.3%), and error trials (5.9%) were excluded as outliers from the RT analysis. The RTs and error rates were submitted to a 4 × 2 repeated-measures ANOVA with the avatar type and position of the avatar and chair as the within-subject factors. If there was a lack of sphericity, the reported values were adjusted using the Greenhouse-Geisser correction [32]. When conducting the multiple comparisons after the ANOVAs, we reported the *p*-values that were corrected using Shaffer’s modified sequentially rejective Bonferroni procedure [33].

### Results

**Fig 8:**
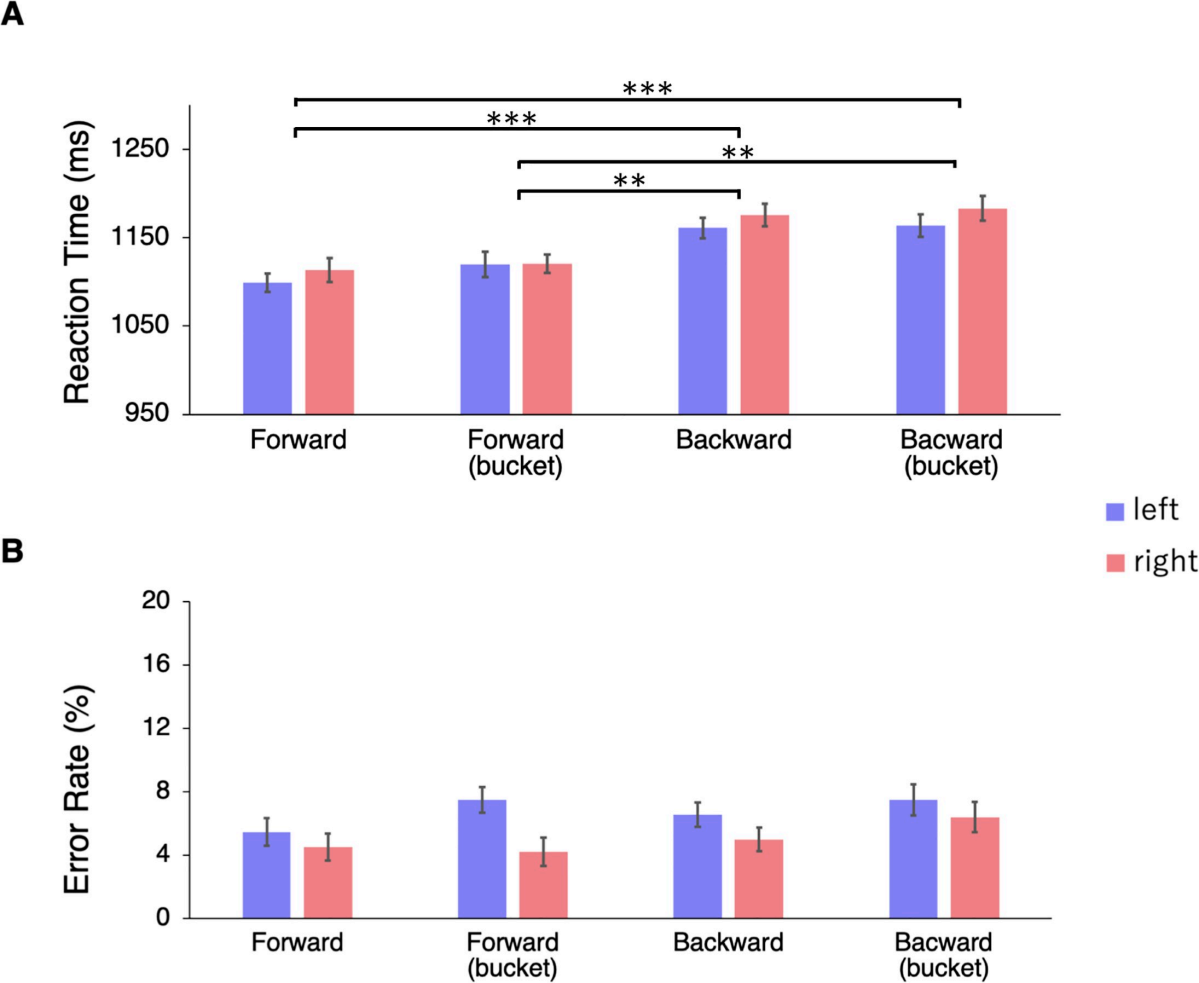
Results of Experiment 4. (A) Mean reaction times (RTs). Two-way repeated measures ANOVA and Shaffer’s post hoc tests were conducted (*p < .05, **p < .01, ***p < .001). The RTs were significantly faster in conditions (1) forward avatar and (2) forward avatar with bucket, than in conditions (3) backward avatar and (4) backward avatar with bucket. (B) Error rates. There were no significant main effects or interactions for the accuracy. Error bars represented 95% within-subject confidence intervals.

The ANOVA of the RTs showed significant main effects of the type of avatar, *F*(3, 57) = 18.005, *p* < 0.001, η_p_^2^ = 0.487. The main effects of the position of the avatar and the interaction were not significant. Post-hoc comparison showed that RTs in condition 1 were shorter than in conditions 3 and 4 (*ps*<0.001), and RTs in condition 2 were also shorter than conditions 3 and 4 (*ps*<0.01) (Fig 8A). There was no difference in RTs between conditions 1 and 2, and between conditions 3 and 4. The ANOVA of error rates revealed that there were no significant main effects or interactions for the accuracy (Fig 8B).

### Discussion

We found that the RTs for the perspective transformation were shorter when the avatar faced the target stimulus (i.e., forward avatar) than when the avatar turned its back on the target (i.e., backward avatar), regardless of whether the avatar’s head was covered with a bucket or not. This indicates that the direction of the torso, not the direction of the head or gaze, was critical for the facilitation effect on visual perspective-taking.

## General discussion

### Summary of results

In Experiment 1, we found that visual perspective transformation was facilitated by a humanoid avatar more than by an empty chair, but only in the short interval condition. The results of Experiment 2 showed that the facilitation of the perspective transformation occurred only when the avatar’s body and head were facing the visual stimulus. Furthermore, in Experiment 3, the facilitation of the perspective transformation diminished when the avatar’s head was oriented in the opposite direction to the torso (i.e., impossible posture). In Experiment 4, the facilitation effect of the forward avatar on perspective transformation lasted even when the avatar’s head was covered with a bucket. This indicates that the direction of the torso is critical, not the gaze or the head direction.

### Embodiment facilitates perspective taking

As mentioned in the introduction, Kessler and Thomson [22] pointed out that while endogenous embodiment is mainly involved in explicit Level 2 perspective taking, the exogenous embodiment of the avatar is helpful for perspective taking. Although the facilitation effect of the presence of the avatar in Level 2 perspective taking has been shown in some previous studies [7, 15], the underlying mechanism was unclear. In this study, we found that the posture of the avatar with respect to the target (especially the direction of the torso) is crucial, and the facilitation effect disappeared in the impossible posture avatar. These results suggest that either spatial attention due to the saliency of the humanoid avatar, drawing of attention by the avatar’s gaze (joint attention), or mentalizing, were not sufficient to explain the facilitation effect. Rather, the results of this study support the effect of the exogenous embodiment [22], that a direct match between the perceived body posture and the observer’s motoric repertoire reduces the time for mental self-rotation.

Our findings are largely consistent with those of Ward et al. [15] and Michelon and Zacks [7]. Ward et al. [15] reported that a humanoid avatar could lead to the participant making a judgment faster if the scene that appeared from the avatar’s position helped when responding to the task, while an inanimate object (a lamp) did not show this effect. However, in their study, it was not clear whether this facilitation effect was due to the saliency of the humanoid avatar or the efficiency of calculating the content of the avatar’s perspective. Michelon and Zacks [7] showed that a Level 2 perspective taking task was performed faster when a doll was presented compared to an asterisk; however, they discussed the cost of the avatar’s absence by comparing two experiments indirectly. In the current study (Experiment 2), we investigated the mechanism that underlies the avatar’s facilitation effect on perspective taking by manipulating only the bodily orientation of the humanoid avatar. As a result, the RT was faster only when the humanoid avatar was facing forward. This suggests that not only spatial attention, but also the embodiment of the avatar, facilitates Level 2 perspective taking.

Furthermore, our results showed that visual perspective taking was not facilitated by the avatar when its posture was biomechanically impossible. Participants’ performance in the impossible-posture avatar condition was worse than in the backward avatar condition. We argue that it was difficult for participants to embody the impossible-posture avatar (see [35]), and therefore the facilitation effect disappeared. Thus, embodiment is a critical factor for the facilitation effect of perspective taking. However, we did not measure sense of embodiment to the avatar. Further investigation on this issue is needed. The RT for the torso-only forward avatar was faster than the head-only forward avatar. This is partly consistent with the study by Ward et al. [16], in which the visual perspective taking occurred even when the avatar diverted its gaze. Furthermore, Longo et al. [36] showed that both the head and torso contribute to perspective taking, but the torso more so than the head. However, our results may not necessarily reflect a difference in the importance of body parts in embodiment to avatars, as an impossible posture avatar is not considered to be embodied [35].

Thus, in Experiment 4, we covered the possible posture avatar’s head with a bucket blocking the eyes to examine which direction of the head or the torso was decisive in the facilitation effect of avatar in Level 2 perspective taking. Results showed that RT for perspective transformation was faster in the forward avatar condition than the backward avatar condition regardless of whether the avatar’s head was covered or not, suggesting that the direction of the torso is important, not the head or gaze direction, for the facilitation effect of the avatar. This is consistent with previous studies that indicated the importance of the torso in perspective taking [16, 36]. In particular, our findings extend the study of Ward et al. [16]. These researchers showed that visual perspective taking occurred spontaneously and facilitated the judgment of identifying the target letter even when the avatar diverted its gaze, concluding that visual perspective taking is a general spatial-navigational ability to the spatial location of another person. However, by introducing the backward avatar (i.e., the whole body is facing away from the target) condition in Experiment 2 and 4, we revealed that the direction of the avatar’s gaze is not essential, but the "correct" direction of the body posture is required for facilitating perspective transformation. These data further support the idea that the embodiment of the avatar underlies the mechanism of facilitation of Level 2 perspective taking.

Regarding Level 1 perspective taking (i.e. whether or not an object can be seen from another person), it is sensitive to the direction of the other person’s line of sight, and blocking the gaze reduces the interference of another person’s perspective (altercentoric intrusion) [13]. Based on this sensitivity for gaze, it is argued that the process of mentalizing, to infer the mental state of what others are seeing, occurs spontaneously in Level 1 perspective taking (for discussion, see also [37, 38]). However, at least in Level 2 perspective taking (i.e., how an object looks), it is unlikely that the "belief" of others’ seeing and the process of "mentalizing" is substantially involved in the facilitation effect of the avatar since blindfolded avatars maintain the effect.

### Speed accuracy trade-off

There was a difference in RT depending on the position of the avatar. In the short interval condition in Experiments 1 and 2, the RTs were longer in the front position than in the right and left positions, and in the long interval condition in Experiment 1, the RTs were longer in the front and right positions than in the left position. The longest RT in the front position obtained in short interval conditions is consistent with previous studies which showed that Level 2 perspective taking involves the process of mental self-rotation to a different viewpoint from one’s own (e.g., [7, 22, 37]). That is, the large angular disparity between the front position and the individual’s own body position required a longer time for mental self-rotation, resulting in a longer RT. In Experiment 1, the main effect of the position was significant (no interaction with other factors) for the error rate, and the performance was significantly accurate in the left and front positions; therefore, the longest RT in the front position can be partly explained by the trade-off with accuracy. However, since Experiment 2 did not show significant main effects or interactions for the accuracy (error rate), the trade-off alone is not a sufficient explanation. The gradient of RT weakened in the long interval condition and there was no difference between the RT in the right position and in the front position. This might be due to the time-gap between the presentation of the avatar or chair and the presentation of the target stimulus. In that case, the observer can finish perspective transformation to any position before presentation of the target stimulus, and therefore, the RT would not differ depending on the avatar’s position. Of course, if this account is reasonable, the result of the left position having a shorter RT than the other two positions appears to be strange, but this may have been due to the sofa presented in the upper left corner of the display. The sofa may have attracted attention and the RT in the left position may have been shortened. The attention capture effect of the sofa may not work under the short interval condition. However, this is just speculation.

### Limitations and future research

There are some limitations to this study. First, the genders of the participants were biased, with the majority being male. As performance differences have been reported for males and females in perspective-taking (e.g., Kessler et al. [39]), we should be cautious about generalizing our results. Second, in Experiments 1, 2, and 3, a sofa was presented at the left end of the room in the virtual environment, and the experiment space was asymmetric. In the short interval condition in Experiment 1 and in Experiments 2 and 3, there was no difference of RTs between left position and right position of the avatar. In Experiment 4, in which the sofa was removed from the virtual room, similar results were obtained showing that the positions of the avatar had no effect on RTs. However, in the long interval condition of Experiment 1, the RTs were fastest when the avatar was in the left position, suggesting the undeniable possibility that spatial attention was attracted to the left side where the sofa was presented. Presumably, the task of this experiment, which causes very rapid embodiment, is unlikely to be affected by spatial attention to the position of the sofa, but it should be considered that the sofa presentation could potentially affect the results. Third, while participants wore a head-mounted display and performed tasks in virtual space, the participant‘s own body was not presented as an avatar in the virtual space. On the other hand, in previous studies showing the embodiment process in Level 2 perspective taking [7, 22], the stimulus was presented on the computer display, allowing participants to see their actual bodies. It might be possible that the inability to see one’s body affects the ease of embodiment of avatars.

This study showed that the direction of a humanoid avatar’s body is crucial for the facilitation effect on the imagined shift of perspective only when the avatar’s posture is biomechanically possible. Thus, the embodiment of the avatar could enhance the imagined shift of perspective. If so, the enhancement should be further improved by illusory ownership to the avatar using the full body illusion (e.g., [40–42]). These points should be examined in future research.

Quesque et al. [43] showed that participants spontaneously take an empty chair’s perspective. Thus, one may argue that the presence of another person is not necessary for visual perspective taking. Our findings are not inconsistent with theirs; we have shown the improvement of visual perspective taking with presenting a humanoid avatar facing the object. Furthermore, Tversky and Hard [21] showed that the frequency of taking the other’s perspective increased when the spatial questions referred to action. Thus, we can speculate that an empty chair may draw participants’ attention to an action, “siting,” or its affordance, and facilitate perspective taking. It is also possible that embodiment was more likely to occur because our task was a motor task using a joystick. Therefore, it is worth investigating whether the visual perspective taking measured by our motor task is facilitated by an empty chair compared to other inanimate objects or a non-affordable chair that we cannot sit in, and by attention to action rather than postural differences. It will further make clear the effect of motoric embodiment on visual perspective taking.
